# 1-{[(*Z*)-Cyclo­pentyl­idene]amino}-3-phenyl­thio­urea

**DOI:** 10.1107/S1600536814007028

**Published:** 2014-04-05

**Authors:** Joel T. Mague, Shaaban K. Mohamed, Mehmet Akkurt, Alaa A. Hassan, Mustafa R. Albayati

**Affiliations:** a Department of Chemistry, Tulane University, New Orleans, LA 70118, USA; b Chemistry and Environmental Division, Manchester Metropolitan University, Manchester M1 5GD, England; c Chemistry Department, Faculty of Science, Minia University, 61519 El-Minia, Egypt; d Department of Physics, Faculty of Sciences, Erciyes University, 38039 Kayseri, Turkey; e Kirkuk University, College of Science, Department of Chemistry, Kirkuk, Iraq

## Abstract

The sample of the title compound, C_12_H_15_N_3_S, chosen for study consisted of triclinic crystals twinned by a 180° rotation about the *a* axis. The five-membered ring adopts a twisted conformation. The dihedral angle between the phenyl ring and the mean plane of the thio­urea unit is 78.22 (8)°. In the crystal, molecules are linked *via* pairs of N—H⋯S hydrogen bonds forming inversion dimers.

## Related literature   

For the use of thio­urea as a building-block in the synthesis of heterocycles, see: Yin *et al.* (2008 ▶). For the diverse biological properties of thio­urea-containing compounds and their metal complexes, see: Saeed *et al.* (2010 ▶); Solomon *et al.* (2010 ▶); Karakuş & Rollas (2002 ▶); Abdullah & Salh (2010 ▶). For the synthesis of the title compound, see: Akkurt *et al.* (2014 ▶). For structural studies on thio­urea derivatives, see: Struga *et al.* (2009 ▶). For ring-puckering parameters, see: Cremer & Pople (1975 ▶).
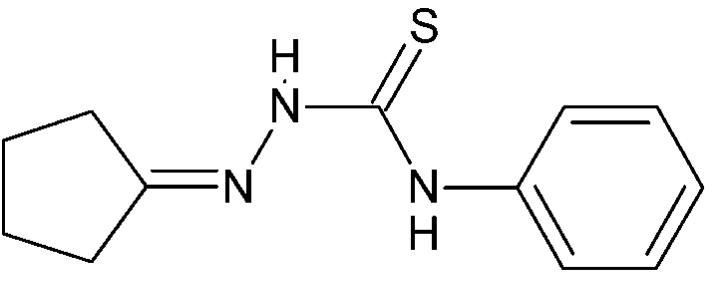


## Experimental   

### 

#### Crystal data  

C_12_H_15_N_3_S
*M* *_r_* = 233.33
Triclinic, 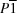

*a* = 7.3997 (2) Å
*b* = 7.5790 (1) Å
*c* = 11.4657 (2) Å
α = 93.0220 (9)°
β = 105.4530 (9)°
γ = 104.7070 (8)°
*V* = 594.45 (2) Å^3^
*Z* = 2
Cu *K*α radiation
μ = 2.21 mm^−1^
*T* = 100 K
0.21 × 0.10 × 0.04 mm

#### Data collection  

Bruker D8 VENTURE PHOTON 100 CMOS diffractometer
Absorption correction: multi-scan (*TWINABS*; Sheldrick, 2009 ▶) *T*_min_ = 0.65, *T*_max_ = 0.92
11363 measured reflections
11360 independent reflections
9454 reflections with *I* > 2σ(*I*)
*R*_int_ = 0.026

#### Refinement  

*R*[*F* ^2^ > 2σ(*F* ^2^)] = 0.040
*wR*(*F* ^2^) = 0.097
*S* = 1.03
11360 reflections
146 parameters
H-atom parameters constrained
Δρ_max_ = 0.28 e Å^−3^
Δρ_min_ = −0.20 e Å^−3^

### 

Data collection: *APEX2* (Bruker, 2013 ▶); cell refinement: *SAINT* (Bruker, 2013 ▶); data reduction: *SAINT* and *CELL_NOW* (Sheldrick, 2008*a* ▶); program(s) used to solve structure: *SHELXS2013* (Sheldrick, 2008*a* ▶); program(s) used to refine structure: *SHELXL2013* (Sheldrick, 2008*a* ▶); molecular graphics: *DIAMOND* (Brandenburg & Putz, 2012 ▶); software used to prepare material for publication: *SHELXTL* (Sheldrick, 2008*a* ▶).

## Supplementary Material

Crystal structure: contains datablock(s) global, I. DOI: 10.1107/S1600536814007028/sj5395sup1.cif

Structure factors: contains datablock(s) I. DOI: 10.1107/S1600536814007028/sj5395Isup2.hkl

CCDC reference: 994387

Additional supporting information:  crystallographic information; 3D view; checkCIF report

## Figures and Tables

**Table 1:** Hydrogen-bond geometry (Å, °)

*D*—H⋯*A*	*D*—H	H⋯*A*	*D*⋯*A*	*D*—H⋯*A*
N2—H2⋯S1^i^	0.91	2.56	3.4636 (18)	172

Symmetry code: (i) 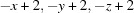.

**Fig. 1.**
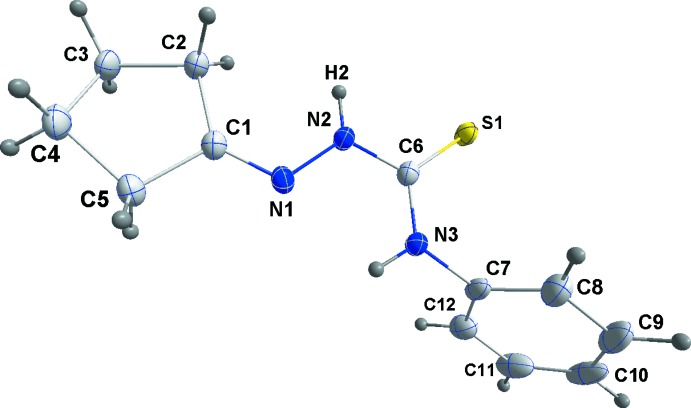
Perspective view of I with 50% probability displacement ellipsoids.

**Fig. 2.**
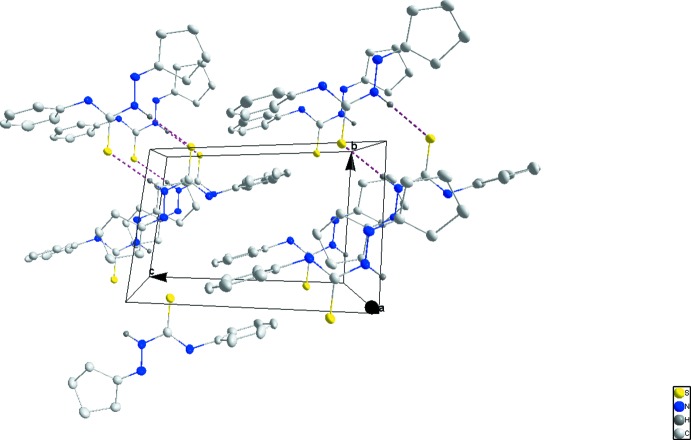
Packing viewed down the a axis and showing N—H···S interactions.

## supplementary crystallographic information

### 1. Comment

For the past few decades, thiourea derivatives have attracted great attention not only because they are important building blocks in the synthesis of heterocycles and organo-metal complexes (Yin *et al.*, 2008) but also due to their broad spectrum of biological activities such as anti-bacterial, anti-cancer (Saeed *et al.*, 2010), anti-malarial (Solomon *et al.*, 2010), anti-tuberculosis (Karakuş & Rollas 2002) anti-convulsion, analgesic and HDL-elevating properties. In addition, metal complex of thiourea derivatives exhibit anti-inflammatory, anti-cancer and anti-fungal activities (Abdullah & Salh, 2010). Furthermore, the thiourea structure contains a central hydrophilic part and two hydrophobic moieties forming a butterfly-like conformation. This conformation is a part of the structure of an anti-HIV agent (Struga *et al.*, 2009).

Fig. 1 shows a perspective view of the title compound (I). The five-membered ring (C1–C5) adopts a *twisted* conformation, [the puckering parameters (Cremer & Pople, 1975) are Q(2) = 0.316 (2) Å and φ(2) = 85.7 (4)°]. The dihedral angle between the phenyl ring and the least-squares plane of the thiourea moiety is 78.22 (8)°.

In the crystal structure, the molecules are connected by weak N—H···S interactions (Fig. 2 and Table 1).

### 2. Experimental

The title compound was prepared according to our previously reported method (Akkurt *et al.*, 2014). Colourless crystals suitable for X-ray diffraction were obtained by crystallization of (I) from ethanol.

### 3. Refinement

H-atoms attached to carbon were placed in calculated positions (C—H = 0.95 - 0.98 Å) while those attached to nitrogen were placed in locations derived from a difference map and their parameters adjusted to give N—H = 0.91 Å. All were included as riding contributions with isotropic displacement parameters 1.2 - 1.5 times those of the attached atoms. The crystal used proved to be twinned by a 180° rotation about *a*, CELL_NOW, (Sheldrick, 2008*a*) and the final structure was refined as a 2-component twin with a refined value for the minor twin fraction of 0.23070 (18).

### Crystal data

**Table:**

C_12_H_15_N_3_S	*Z* = 2
*M**_r_* = 233.33	*F*(000) = 248
Triclinic, *P*1	*D*_x_ = 1.304 Mg m^−^^3^
*a* = 7.3997 (2) Å	Cu *K*α radiation, λ = 1.54178 Å
*b* = 7.5790 (1) Å	Cell parameters from 8773 reflections
*c* = 11.4657 (2) Å	θ = 4.0–70.0°
α = 93.0220 (9)°	µ = 2.21 mm^−^^1^
β = 105.4530 (9)°	*T* = 100 K
γ = 104.7070 (8)°	Plate, colourless
*V* = 594.45 (2) Å^3^	0.21 × 0.10 × 0.04 mm

### Data collection

**Table:**

Bruker D8 VENTURE PHOTON 100 CMOS diffractometer	11360 independent reflections
Radiation source: INCOATEC IµS micro–focus source	9454 reflections with *I* > 2σ(*I*)
Mirror monochromator	*R*_int_ = 0.026
Detector resolution: 10.4167 pixels mm^-1^	θ_max_ = 70.0°, θ_min_ = 4.0°
ω scans	*h* = −8→8
Absorption correction: multi-scan (*TWINABS*; Sheldrick, 2009)	*k* = −9→9
*T*_min_ = 0.65, *T*_max_ = 0.92	*l* = −13→13
11363 measured reflections	

### Refinement

**Table:**

Refinement on *F*^2^	Primary atom site location: structure-invariant direct methods
Least-squares matrix: full	Secondary atom site location: difference Fourier map
*R*[*F*^2^ > 2σ(*F*^2^)] = 0.040	Hydrogen site location: mixed
*wR*(*F*^2^) = 0.097	H-atom parameters constrained
*S* = 1.03	*w* = 1/[σ^2^(*F*_o_^2^) + (0.039*P*)^2^ + 0.1956*P*] where *P* = (*F*_o_^2^ + 2*F*_c_^2^)/3
11360 reflections	(Δ/σ)_max_ = 0.001
146 parameters	Δρ_max_ = 0.28 e Å^−^^3^
0 restraints	Δρ_min_ = −0.20 e Å^−^^3^

### Special details

**Table:**

Experimental. Analysis of 985 reflections having I/σ(I) > 15 and chosen from the full data set with *CELL_NOW* (Sheldrick, 2008*a*) showed the crystal to belong to the triclinic system and to be twinned by a 180° rotation about the *a* axis. The raw data were processed using the multi-component version of *SAINT* under control of the two-component orientation file generated by *CELL_NOW*.
Geometry. All e.s.d.'s (except the e.s.d. in the dihedral angle between two l.s. planes) are estimated using the full covariance matrix. The cell e.s.d.'s are taken into account individually in the estimation of e.s.d.'s in distances, angles and torsion angles; correlations between e.s.d.'s in cell parameters are only used when they are defined by crystal symmetry. An approximate (isotropic) treatment of cell e.s.d.'s is used for estimating e.s.d.'s involving l.s. planes.
Refinement. Refinement of *F*^2^ against ALL reflections. The weighted *R*-factor *wR* and goodness of fit *S* are based on *F*^2^, conventional *R*-factors *R* are based on *F*, with *F* set to zero for negative *F*^2^. The threshold expression of *F*^2^ > σ(*F*^2^) is used only for calculating *R*-factors(gt) *etc*. and is not relevant to the choice of reflections for refinement. *R*-factors based on *F*^2^ are statistically about twice as large as those based on *F*, and *R*- factors based on ALL data will be even larger. H-atoms attached to carbon were placed in calculated positions (C—H = 0.95 - 0.99 Å) while those attached to nitrogen were placed in locations derived from a difference map and their parameters adjusted to give N—H = 0.91 Å. All were included as riding contributions with isotropic displacement parameters 1.2 times those of the attached atoms. Refined as a 2-component twin.

### Fractional atomic coordinates and isotropic or equivalent isotropic displacement parameters (Å^2^)

**Table:**

	*x*	*y*	*z*	*U*_iso_*/*U*_eq_	
S1	0.89113 (8)	1.01459 (7)	0.81488 (4)	0.02372 (18)	
N1	0.7313 (2)	0.5215 (2)	0.90890 (15)	0.0216 (4)	
N2	0.8223 (3)	0.7082 (2)	0.91604 (15)	0.0213 (4)	
H2	0.8859	0.7824	0.9880	0.026*	
N3	0.7179 (2)	0.6693 (2)	0.70759 (14)	0.0226 (4)	
H3	0.6668	0.5483	0.7126	0.027*	
C1	0.7350 (3)	0.4552 (3)	1.01002 (18)	0.0201 (5)	
C2	0.8303 (3)	0.5512 (3)	1.13816 (17)	0.0221 (5)	
H2A	0.7989	0.6695	1.1480	0.027*	
H2B	0.9739	0.5749	1.1600	0.027*	
C3	0.7435 (3)	0.4160 (3)	1.21708 (19)	0.0274 (5)	
H3A	0.8400	0.4244	1.2973	0.033*	
H3B	0.6258	0.4424	1.2306	0.033*	
C4	0.6913 (3)	0.2257 (3)	1.14529 (19)	0.0280 (5)	
H4A	0.5820	0.1399	1.1656	0.034*	
H4B	0.8049	0.1748	1.1635	0.034*	
C5	0.6317 (3)	0.2559 (3)	1.01077 (19)	0.0242 (5)	
H5A	0.6742	0.1736	0.9604	0.029*	
H5B	0.4885	0.2326	0.9791	0.029*	
C6	0.8052 (3)	0.7858 (3)	0.81110 (18)	0.0201 (5)	
C7	0.6780 (3)	0.7262 (3)	0.58782 (18)	0.0240 (5)	
C8	0.8249 (4)	0.7674 (3)	0.5318 (2)	0.0358 (6)	
H8	0.9527	0.7619	0.5729	0.043*	
C9	0.7831 (5)	0.8171 (4)	0.4146 (2)	0.0453 (7)	
H9	0.8827	0.8458	0.3751	0.054*	
C10	0.5971 (5)	0.8248 (3)	0.3557 (2)	0.0448 (7)	
H10	0.5690	0.8582	0.2755	0.054*	
C11	0.4526 (4)	0.7845 (3)	0.4123 (2)	0.0410 (7)	
H11	0.3249	0.7906	0.3714	0.049*	
C12	0.4930 (4)	0.7346 (3)	0.5295 (2)	0.0309 (5)	
H12	0.3933	0.7066	0.5690	0.037*	

### Atomic displacement parameters (Å^2^)

**Table:**

	*U*^11^	*U*^22^	*U*^33^	*U*^12^	*U*^13^	*U*^23^
S1	0.0326 (3)	0.0182 (3)	0.0185 (3)	0.0051 (2)	0.0058 (2)	0.0045 (2)
N1	0.0248 (10)	0.0173 (9)	0.0231 (9)	0.0043 (8)	0.0089 (8)	0.0044 (7)
N2	0.0278 (10)	0.0178 (9)	0.0159 (9)	0.0025 (7)	0.0058 (7)	0.0029 (7)
N3	0.0303 (10)	0.0178 (9)	0.0165 (9)	0.0016 (8)	0.0056 (8)	0.0036 (7)
C1	0.0203 (11)	0.0204 (11)	0.0229 (11)	0.0082 (9)	0.0091 (9)	0.0057 (9)
C2	0.0244 (11)	0.0220 (11)	0.0208 (11)	0.0067 (9)	0.0070 (9)	0.0062 (9)
C3	0.0293 (12)	0.0298 (12)	0.0225 (11)	0.0056 (10)	0.0078 (10)	0.0104 (9)
C4	0.0264 (12)	0.0257 (12)	0.0333 (13)	0.0064 (10)	0.0097 (10)	0.0135 (10)
C5	0.0263 (12)	0.0198 (11)	0.0276 (12)	0.0053 (9)	0.0104 (10)	0.0053 (9)
C6	0.0197 (11)	0.0235 (11)	0.0196 (10)	0.0076 (9)	0.0076 (9)	0.0062 (9)
C7	0.0369 (13)	0.0156 (10)	0.0161 (10)	0.0031 (10)	0.0064 (10)	0.0015 (8)
C8	0.0437 (15)	0.0386 (14)	0.0242 (12)	0.0061 (12)	0.0133 (11)	0.0051 (10)
C9	0.073 (2)	0.0360 (14)	0.0241 (13)	−0.0004 (14)	0.0238 (14)	0.0033 (11)
C10	0.088 (2)	0.0192 (12)	0.0158 (12)	0.0041 (13)	0.0058 (14)	0.0039 (9)
C11	0.0595 (18)	0.0246 (13)	0.0279 (13)	0.0113 (12)	−0.0054 (13)	0.0043 (10)
C12	0.0402 (15)	0.0224 (11)	0.0269 (12)	0.0075 (11)	0.0054 (11)	0.0040 (9)

### Geometric parameters (Å, º)

**Table:**

S1—C6	1.682 (2)	C4—C5	1.533 (3)
N1—C1	1.284 (2)	C4—H4A	0.9900
N1—N2	1.392 (2)	C4—H4B	0.9900
N2—C6	1.357 (2)	C5—H5A	0.9900
N2—H2	0.9098	C5—H5B	0.9900
N3—C6	1.341 (3)	C7—C12	1.373 (3)
N3—C7	1.439 (2)	C7—C8	1.382 (3)
N3—H3	0.9098	C8—C9	1.391 (3)
C1—C2	1.503 (3)	C8—H8	0.9500
C1—C5	1.512 (3)	C9—C10	1.379 (4)
C2—C3	1.535 (3)	C9—H9	0.9500
C2—H2A	0.9900	C10—C11	1.373 (4)
C2—H2B	0.9900	C10—H10	0.9500
C3—C4	1.526 (3)	C11—C12	1.391 (3)
C3—H3A	0.9900	C11—H11	0.9500
C3—H3B	0.9900	C12—H12	0.9500
			
C1—N1—N2	117.12 (17)	C1—C5—C4	104.55 (17)
C6—N2—N1	118.43 (17)	C1—C5—H5A	110.8
C6—N2—H2	118.3	C4—C5—H5A	110.8
N1—N2—H2	123.1	C1—C5—H5B	110.8
C6—N3—C7	123.96 (16)	C4—C5—H5B	110.8
C6—N3—H3	118.7	H5A—C5—H5B	108.9
C7—N3—H3	116.9	N3—C6—N2	115.83 (18)
N1—C1—C2	128.72 (18)	N3—C6—S1	123.58 (14)
N1—C1—C5	120.66 (18)	N2—C6—S1	120.59 (16)
C2—C1—C5	110.61 (16)	C12—C7—C8	120.89 (19)
C1—C2—C3	104.01 (17)	C12—C7—N3	119.41 (19)
C1—C2—H2A	111.0	C8—C7—N3	119.68 (19)
C3—C2—H2A	111.0	C7—C8—C9	119.1 (2)
C1—C2—H2B	111.0	C7—C8—H8	120.4
C3—C2—H2B	111.0	C9—C8—H8	120.4
H2A—C2—H2B	109.0	C10—C9—C8	120.0 (3)
C4—C3—C2	105.41 (17)	C10—C9—H9	120.0
C4—C3—H3A	110.7	C8—C9—H9	120.0
C2—C3—H3A	110.7	C11—C10—C9	120.4 (2)
C4—C3—H3B	110.7	C11—C10—H10	119.8
C2—C3—H3B	110.7	C9—C10—H10	119.8
H3A—C3—H3B	108.8	C10—C11—C12	120.0 (2)
C3—C4—C5	105.07 (16)	C10—C11—H11	120.0
C3—C4—H4A	110.7	C12—C11—H11	120.0
C5—C4—H4A	110.7	C7—C12—C11	119.6 (2)
C3—C4—H4B	110.7	C7—C12—H12	120.2
C5—C4—H4B	110.7	C11—C12—H12	120.2
H4A—C4—H4B	108.8		
			
C1—N1—N2—C6	−173.97 (17)	N1—N2—C6—N3	−6.8 (3)
N2—N1—C1—C2	−1.9 (3)	N1—N2—C6—S1	173.42 (14)
N2—N1—C1—C5	177.10 (17)	C6—N3—C7—C12	−100.6 (2)
N1—C1—C2—C3	166.7 (2)	C6—N3—C7—C8	80.9 (3)
C5—C1—C2—C3	−12.4 (2)	C12—C7—C8—C9	−0.3 (3)
C1—C2—C3—C4	27.7 (2)	N3—C7—C8—C9	178.2 (2)
C2—C3—C4—C5	−32.9 (2)	C7—C8—C9—C10	0.0 (4)
N1—C1—C5—C4	173.24 (18)	C8—C9—C10—C11	0.3 (4)
C2—C1—C5—C4	−7.6 (2)	C9—C10—C11—C12	−0.3 (4)
C3—C4—C5—C1	24.8 (2)	C8—C7—C12—C11	0.3 (3)
C7—N3—C6—N2	176.88 (18)	N3—C7—C12—C11	−178.16 (19)
C7—N3—C6—S1	−3.4 (3)	C10—C11—C12—C7	0.0 (3)

### Hydrogen-bond geometry (Å, º)

**Table:**

*D*—H···*A*	*D*—H	H···*A*	*D*···*A*	*D*—H···*A*
N2—H2···S1^i^	0.91	2.56	3.4636 (18)	172

Symmetry code: (i) −*x*+2, −*y*+2, −*z*+2.
